# Changes in choroidal vascular structure from vitreoretinal lymphoma and the intraocular cytokine level associated with clinical resolution after intravitreal methotrexate treatment

**DOI:** 10.1371/journal.pone.0260469

**Published:** 2021-11-23

**Authors:** Rae-Young Kim, Jae Hyun Park, Mirinae Kim, Young-Geun Park, Seok-Goo Cho, Young-Hoon Park

**Affiliations:** 1 Department of Ophthalmology and Visual Science, Seoul St. Mary’s Hospital, College of Medicine, The Catholic University of Korea, Seoul, Korea; 2 Catholic Institute for Visual Science, College of Medicine, The Catholic University of Korea, Seoul, Korea; 3 Department of Hematology, Seoul St. Mary’s Hospital, College of Medicine, The Catholic University of Korea, Seoul, Korea; Massachusetts Eye & Ear Infirmary, Harvard Medical School, UNITED STATES

## Abstract

**Purpose:**

To evaluate changes in choroidal vascular structure and aqueous cytokine levels in eyes with vitreoretinal lymphoma (VRL) after intravitreal methotrexate (MTX) treatment.

**Methods:**

In this retrospective study, VRL patients who visited our hospital between October 2018 and July 2020 were reviewed. Aqueous samples were obtained before treatment and at clinical resolution after intravitreal MTX therapy. Interleukin (IL)-6 and IL-10 levels and the IL-10-to-IL-6 ratio were evaluated. Swept-source optical coherence tomographic images were obtained along with the aqueous samples. Subfoveal choroidal thickness (SFCT), total vascular area of the choroid (TCA), stromal area (SA), luminal area (LA), and choroidal vascularity index (CVI) were assessed.

**Results:**

Twelve patients were enrolled (female:male—5:7). The mean age (± standard deviation) at diagnosis was 60.9±8.5 years. In the 16 eyes diagnosed with VRL, values of SFCT, TCA, LA, and SA significantly decreased after treatment (all p-values <0.05). Additionally, the aqueous cytokine IL-10 level and IL-10-to-IL-6 ratio were significantly decreased (p = 0.001 and p = 0.003, respectively). The choroidal structure in the non-treated fellow eyes did not show any significant difference. There were no further changes in SFCT, TCA, LA, or CVI that occurred during maintenance therapy. For clinical remission, the patients received 7.7±5.5 intravitreal MTX injections. The required number of injections for clinical remission was positively correlated with best-corrected visual acuity, IL-10, and IL-6 levels in the active phase (p = 0.035, p = 0.009, and p = 0.031, respectively).

**Conclusion:**

Eyes with active VRL exhibited choroidal thickening with increased vascular and stromal areas that decreased after remission following MTX treatment. Higher aqueous IL-10 and IL-6 levels and lower visual acuity in the active phase may indicate the number of injections required for remission; this should be considered in the treatment of patients with VRL.

## Introduction

Vitreoretinal lymphoma (VRL) is a rare subtype of central nervous system lymphoma [1, 2]. The most common histopathologic type of VRL is diffuse large B-cell lymphoma (DLBCL). Additionally, it is the most common subtype of non-Hodgkin’s lymphoma [3]. Approximately 65–90% of patients with primary VRL (PVRL) develop accompanying central nervous system (CNS) lymphomas over time, while over 15% of primary CNS lymphoma patients develop accompanying intraocular lymphoma [1, 4]. The five-year cumulative survival rates are 35% and 68%, respectively, when accompanied or not accompanied by CNS extension, emphasizing that CNS involvement worsens the prognosis. In one study of 47 patients with PVRL, the five-year survival rate was 41.4%, and the median overall survival was 38 months, which was poor compared to DLBCL that occurs outside the central nervous system [5, 6]. If ophthalmologic ocular symptoms develop first, prompt diagnosis is important to prevent delays in systemic assessment and treatment. VRL patients often complain of non-specific symptoms, such as blurred vision (40–50%), reduced visual acuity (25–30%), and floaters (20–25%). Further complicating the diagnosis is the need to differentiate VRL from several other conditions, including infectious and non-infectious uveitis [1, 7]. Moreover, VRL diagnosis requires histopathological identification of malignant lymphoid cells from vitreous or retinal samples, but cytology alone has a high false-positive ratio due to fragility, loss, and damage of tumor cells, and limited quantities of ocular samples. Therefore, a multidisciplinary approach is required to increase the diagnostic rate [3].

Additionally, interleukin (IL)-10 is a cytokine derived from malignant lymphoma cells that can evade B-cell antibody production and cellular immunity. Compared to IL-12 and IL-6, which are known to increase in general intraocular inflammatory states, in VRL patients, IL-10 shows specifically elevated levels in the vitreous and aqueous humor (AH) [4, 8, 9]. Elevation of IL-10 above 30–50 pg/mL in the AH and 65–400 pg/mL in the vitreous humor or an IL-10-to-IL-6 ratio >1 in the AH are indicative of VRL [10, 11]. Furthermore, the IL-10 level in the AH decreases with treatment and increases during disease recurrence, indicating that it can be used as an indicator of treatment response [12].

Multimodal non-invasive imaging modalities help with diagnosis and are important for verifying treatment response as well as in testing for recurrence [13, 14]. Optical coherence tomography (OCT) findings in patients with VRL include hyper-reflective material in the inner layer of the retina, in the sub-retina, or in the sub-retinal pigment epithelial (RPE) space [15, 16]. Additionally, well defined hyper-reflective nodules, vertical hyper-reflective band, undulation of RPE, pigment epithelial detachment (PED), sub-retinal fluid (SRF), macular edema (ME), epiretinal membrane (ERM), and sub-retinal fibrosis may be found in OCT of vitreoretinal lymphoma [15–17]. OCT findings other than ERM due to secondary gliosis and sub-retinal fibrosis indicate improvements after treatment and thus help in assessing treatment response [18].

Cicinelli et al. analyzed multimodal images to investigate retinal or choroidal changes after VRL treatment and reported decreased choroidal thickness after intravitreal rituximab injection, but there is still a lack of information regarding structural changes in the choroid following treatment, or their clinical significance [19]. Hence, in this study, we used binarization of OCT images to analyze specific changes in the choroidal structure following intravitreal injection in patients with VRL. Additionally, we examined the correlation between changes in cytokine levels, which is another factor related to lymphoma progression.

## Materials and methods

### Study design

We enrolled patients with a verified diagnosis of VRL who received treatment at the Department of Ophthalmology and Visual Science at Seoul St Mary’s Hospital of the Catholic University of Korea, which is a tertiary medical institution, between October 2018 and July 2020. We also conducted a retrospective review of their medical records. The study protocol adhered to the tenets of the Declaration of Helsinki and was approved by the Catholic University of Korea, Seoul St Mary’s Hospital, Institutional Review Board (KC20RISI0955). The need to obtain informed consent was waived because of the retrospective nature of the study.

Sixteen eyes of 12 patients diagnosed with VRL were enrolled in this study. Patients were diagnosed by two senior attending retinal specialists (Y.H.P. and Y.G.P.), identified by searching their medical records.

Diagnosis of VRL was based on the typical clinical features and cytokine level results. A combination of IL-10-to-IL-6 ratios and total IL-10 concentrations with the distinct clinical pattern of VRL can provide an accurate diagnosis, even if histopathological confirmation is lacking [20–22]. Once diagnosed, VRL patients were referred to a hematologist for evaluation of systemic and CNS involvement. Patients diagnosed with other pathologic vitreoretinal diseases, other known causes of infectious or non-infectious uveitis, and uncontrolled glaucoma were excluded. For wash-out of the effects from other drugs and previous treatment, patients treated with intravitreal steroid or intravitreal methotrexate and/or with a history of vitrectomy within six months from the start of the study were excluded. Cases without appropriate imaging data or cytokine sampling were also excluded.

We reviewed and collected patient data, including sex, age, history of general medical records, systemic chemotherapy and its response, ophthalmologic history, ocular examinations including best-corrected visual acuity (BCVA), non-contact pneumatic tonometry, slit-lamp biomicroscopy, and dilated fundoscopy, and analyzed findings from multimodal imaging including swept-source OCT (SS-OCT) and fundus photography. BCVA measured using the Snellen chart was converted to a corresponding logarithm of the minimum angle of resolution scale value for statistical analysis.

An SS-OCT device (DRI Triton; Topcon, Tokyo, Japan) was used for the choroidal structural evaluation. This device, equipped with a 1,050-nm wavelength light source, can perform 100,000 A-scans/s as per the manufacturer’s statement. OCT examination was performed on the same day as the intravitreal injection, and OCT of non-treated fellow eyes was also collected for comparison. However, if there was no image of appropriate quality for analysis or other retinal diseases that may affect the test result were present in the opposite eye, data for both eyes were excluded from the analysis.

All patients received ocular treatment with a 400 μg/0.1 mL MTX intravitreal injection, which was performed according to the following therapeutic regimen: in the first month, MTX was injected into the vitreous cavity twice per week, followed by weekly injections for one month, and then by monthly maintenance injections. If a patient had a relapse during monthly maintenance therapy, the injection interval was shortened by weekly treatment. If a patient had a MTX related corneal epitheliopathy, the treatment interval was extended early than regimen, from twice-weekly to once-weekly injections or from weekly to monthly treatment.

Before the first MTX injection, a 0.1 mL AH sample was obtained through the limbal margin using a 30-gauge needle. Cytokine measurement of the aqueous sample was repeated when the clinical condition of the study eye satisfied the following criteria for clinical remission: the resolution of previous pathologic clinical findings, including the absence of cells in the anterior chamber and vitreous and disappearance of fundus findings, such as hyper-reflective foci in the subretinal or posterior vitreous, sub-RPE deposit or RPE undulation, and retinal or subretinal infiltration [23].

### Image analysis

Each patient underwent OCT examination on the same day as their aqueous cytokine examination. Choroidal measurements from OCT images were performed by two experienced independent retinal specialists (J.Y.K. and J.H.K.) who were blinded to the patients’ clinical history and other imaging findings.

Subfoveal choroidal thickness (SFCT) was measured manually at the foveal center using an automatic digital caliper built into the software of the SS-OCT device. SFCT was defined as the distance from the outer border of the RPE to the inner edge of the suprachoroidal space.

We selected a raster scan passing through the fovea for analysis to standardize the OCT scan areas and control for natural variation between scans; the fovea-centered 1,500 μm width (750 μm on either side of the fovea) area was measured for the total choroidal area (TCA). TCA and the luminal area (LA) and stromal area (SA) were measured using Image J software version 1.53 (US NIH, Bethesda, MD, USA).

**Fig 1:**
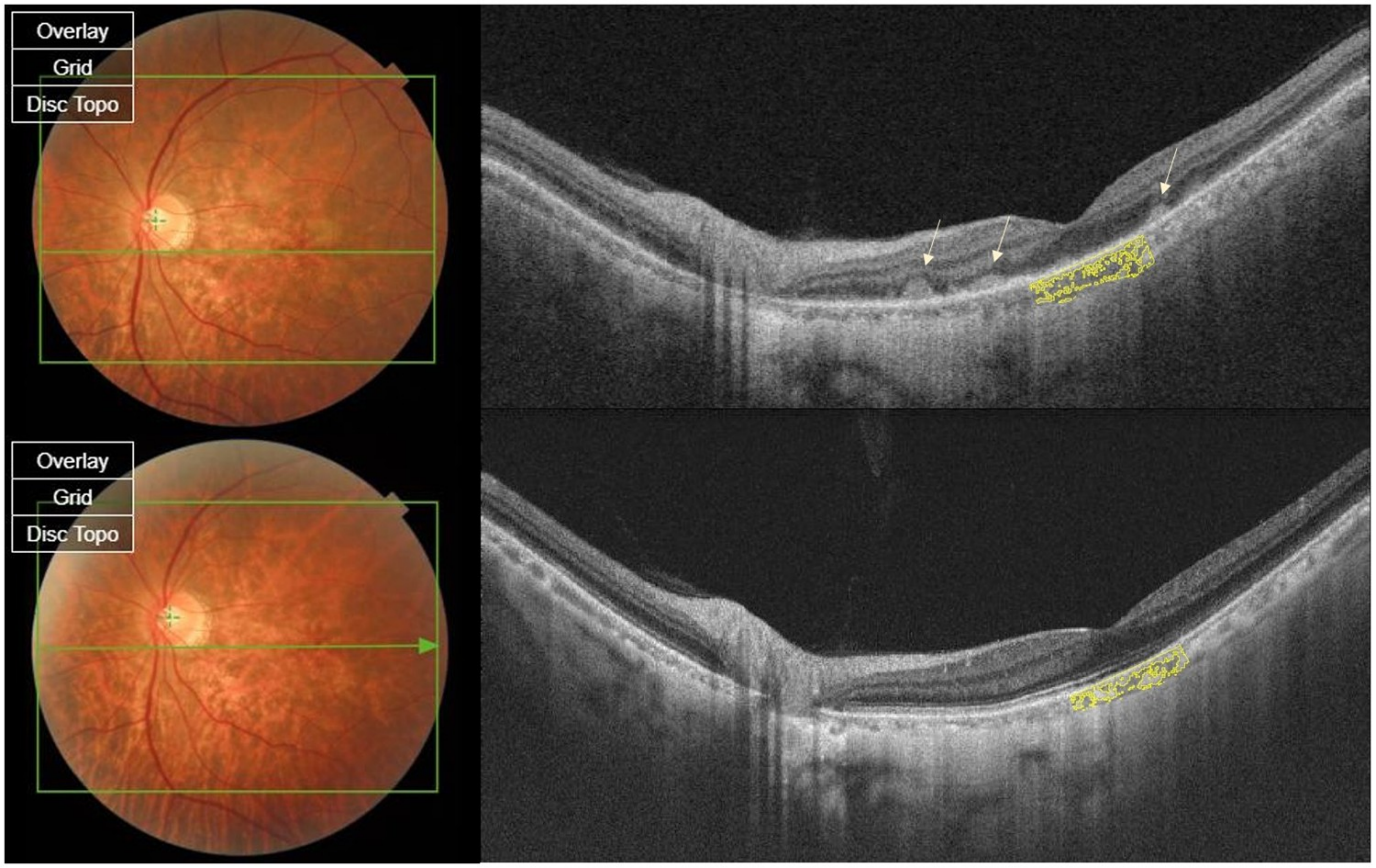
Fundus photo and optical coherence tomography (OCT) scans of a 54-year-old male patient diagnosed with vitreoretinal lymphoma in his left eye. (A) A fundus photo in the active phase (before methotrexate treatment) shows multiple whitish lesions on the macula, and this appears as subretinal infiltration on OCT (arrow). OCT shows obliteration of the sub-retinal pigment epithelium (RPE) and outer retinal hyper-reflective layer. Aqueous cytokine level analysis shows elevation in interleukin (IL)-10 (1720.0 pg/mL) and in the IL-10-to-IL-6 ratio (1720). (B) In the remission phase, the fundus photo shows loss of the whitish lesions, and OCT shows the absence of infiltration with the recovery of the RPE and outer-retinal layer. The sub-foveal choroidal thickness and choroidal area are decreased compared to that in the active phase. The IL-10 level (27.8 pg/mL) and IL-10-to-IL-6 ratio (5.35) have also decreased.

Choroidal LA and SA were measured using the image binarization process described by Agrawal et al. [23] (Fig 1). The selected TCA with a polygonal tool was converted to an 8-bit image. A color threshold was applied after applying the Niblack auto local threshold tool to obtain the mean pixel value with the standard deviation (SD) for all points. The SA highlighted through this process was added to the region of interest (ROI) manager. TCA and SA were merged through “AND” processed in ROI manager, then added to the ROI manager as the third area. The LA in the polygon was obtained by subtracting the SA from the total polygon area and represented the vascular area of the choroid. The ratio of LA to TCA was defined as the choroidal vascularity index (CVI).

The TCA was defined as the area between the outer border of the RPE and the inner edge of the suprachoroidal interface. To assess the CVI, the raster scan, which passes through the fovea, was selected and processed as described by Agrawal et al. [24], and the image binarization process was performed using ImageJ software (version 1.53; https://imagej.nih.gov/ij/).

### Aqueous cytokine level

Before the primary intravitreal MTX injection was performed, 0.1 mL of AH was obtained using a 30-gauge needle with a 1 mL syringe. Cytokine levels were measured in undiluted aqueous samples using a bead-based assay run on a LUMINEX MAGPIX system (Luminex Corp., Austin, TX, USA).

### Statistical analysis

The Statistical Package for the Social Sciences for Windows version 24.0 (SPSS, Inc., Chicago, IL, USA) was used for all statistical analyses. An exploratory analysis was conducted for all variables. The normality of the data distribution was confirmed using the Kolmogorov-Smirnov test. According to the normality test results, data were assessed using either a paired *t*-test, Wilcoxon signed-rank test, or Kruskal–Wallis test. Pearson’s correlation test and Spearman’s rho test were used for evaluating correlations among clinical values. The level for statistical significance was set at p<0.05.

## Results

Initially, the records of 84 eyes from 50 patients were reviewed, and 16 eyes from 12 patients were finally enrolled for analysis (Table 1). Fifty eyes were excluded due to inadequate OCT quality or missing OCT exam for analysis, 12 eyes were lost during follow-up, and in 6 eyes, intravitreal therapy was stopped due to deterioration of the systemic disease. Cytopathology revealed DLBCL in 12 eyes (75%) versus B-cell clonal expansion in 3 eyes (18.8%); the latter was verified by immunoglobulin heavy chain (IgH) gene rearrangement. In 1 eye (6.3%), the histopathologic origin of the malignancy was not identified. Five were female (42%), and seven were male (58%). The mean age at diagnosis was 61.5±8.5 (mean ± SD) years. During follow-up, seven patients (58%) developed CNS involvement of lymphoma. Systemic high-dose MTX treatment was administered to seven patients. Three of the seven patients achieved systemic complete remission (CR), which was maintained after the cessation of systemic treatment. The other four had a partial response (PR) to the treatment.

**Table 1:** Demographic and clinical characteristics of the participants.

Patient No.	Age (years)	Sex	OD/OS	CNS or systemic involvement	IL-10 level at active phase (pg/mL)	IL-6 level at active phase (pg/mL)	Initial IL-10/IL-6 ratio	No. of intravitreal MTX treatments	Result of biopsy	Primary lesion	Systemic CTx	Result of systemic CTx
1	54	F	OD	+, Brain	2105.00	1372.00	1.53	7	DLBCL	Brain with eye	HD-MTX	CR
			OS		5087.00	54.90	92.66	7				
2	64	F	OS	+, Brain	5231.00	100.30	52.15	8	DLBCL	Brain with eye	HD-MTX	CR
3	57	M	OD	+ Brain	917.20	1.70	539.53	4	DLBCL	Eye	HD-MTX	Relapse
			OS	-	9149.00	22.80	401.27	6				
4	54	F	OS	-	613.40	5.50	111.53	3	IgH gene rearrangement	Eye	None	
5	78	M	OS	-	33277.00	122.70	271.21	13	IgH gene rearrangement	Eye	None	
6	54	M	OD	+, Brain	16107.00	554.70	29.04	27	DLBCL	Eye	HD-MTX	Relapse
			OS		1720.00	1.00	1720.00	7				
7	71	M	OD	+, Brain	267.50	176.70	1.51	7	DLBCL	Eye	HD-MTX	PR, poor general condition
			OS		248.10	62.20	3.99	5				
8	74	F	OD	-	1479.00	3.20	462.19	5	Non-defined	Eye	None	
9	63	M	OS	-	568.80	88.50	6.43	4	IgH gene rearrangement	Eye	None	
10	63	M	OS	-	8170.00	10.00	817.00	5	DLBCL	Eye	HD-MTX	
11	53	M	OD	+, Brain	4672.00	62.20	75.11	7	DLBCL	Brain with eye	HD-MTX	PR
12	53	F	OD	+, Brain	2160.00	12.20	177.05	8	DLBCL	Eye	HD-MTX	PR

CNS, central nervous system; F/U, follow up; IL-6, aqueous interleukin 6 level; IL-10, aqueous interleukin-10 level; IL-10/6 ratio, ratio calculated by the IL-10 level divided by the IL-6 level; No, number; OD, oculus dexter; OS, oculus sinister; MTX, methotrexate; CTx, chemotherapy; DLBCL, diffuse large B-cell lymphoma; IgH, immunoglobulin heavy chain; HD-MTX, systemic high dose methotrexate; CR, complete remission; PR, partial remission.

Four of the 12 patients presented with bilateral active VRL (33%). The mean BCVA at baseline was 0.54±0.44 (logMAR). For clinical remission, the patients received a mean number of 7.7±5.5 intravitreal MTX injections. There were no complications following intravitreal injection, including elevation of intraocular pressure (IOP) >21 mm Hg, intraocular hemorrhage, or endophthalmitis.

### Cytokine levels in the aqueous humor

Aqueous cytokine levels were obtained for all 16 eyes before treatment and during the remission phase. Remission cytokine sampling was performed at 40.6 ± 61.4 (mean ± SD) after initial treatment. At baseline, the levels of aqueous IL-6 and IL-10 were elevated in all eyes (Table 1), and the mean values were 5735.8±2123.1 pg/mL and 165.7±87.2 pg/mL, respectively. In addition, the IL-10-to-IL-6 ratio was over 1.0 in all 16 eyes, with a mean value of 297.6±112.0 (Table 2).

**Table 2:** Comparison of clinical parameters at baseline (pre-treatment) and the point of clinical resolution after intravitreal methotrexate injection.

		SFCT	TCA	LA	SA	CVI	BCVA	IL-10	IL-6	IL-10/IL-6 ratio
Study eye	Active phase	290.8 ± 93.5	1.30 ± 1.79	0.98 ± 1.80	0.32 ± 0.08	63.5 ± 12.6	0.54 ± 0.44	5735.8 ± 8492.6 (2132.5)	165.7 ± 349.0 (58.6)	297.6 ± 447.9 (102.1)
(n = 16)	Remission phase	244.4 ± 86.2	0.69 ± 0.23	0.45 ± 0.22	0.26 ± 0.06	61.2 ± 6.8	0.41 ± 0.49	1755.0 ± 6545.5 (2.9)	849.2 ± 3143.6 (10.6)	19.2 ± 48.1 (0.5)
	p-value	<0.001*	0.001*	0.004*	0.025*	0.390	0.176	0.001*	0.733	0.003*
Non treated fellow eye	Active phase	241.3 ± 100.9	0.94 ± 0.37	0.60 ± 0.25	0.34 ± 0.12	63.21 ± 3.83				
(n = 7)	Remission phase	244.4 ± 103.1	0.92 ± 0.36	0.57 ± 0.23	0.35 ± 0.13	61.48 ± 2.35				
	p-value	0.204	0.128	0.611	0.091	0.237				

Pre-treatment was defined as the point before intravitreal methotrexate injection, and post-treatment was defined as the point at which the treated eye met the clinical remission criteria.

Data are expressed as mean ± standard deviation (median); TCA, LA, and SA in mm^2^; CVI in %; BCVA in logMAR; SFCT in μm; IL-10 and IL-6 in pg/mL; IL-10/6 ratio, obtained by dividing the value of IL-10 by the IL-6 value.

TOE, non-treated eyes; SFCT, subfoveal choroidal thickness; TCA, total choroidal area; LA, luminal area; SA, stromal area; CVI, choroidal vascularity index; BCVA, best-corrected visual acuity; logMAR, minimal angle of resolution; IL, interleukin.

A paired *t*-test was used for factors that satisfied normality, and the Wilcoxon signed-rank test was performed for factors that did not satisfy normality.

*p <0.05 were considered to be statistically significant.

The changes in aqueous cytokine levels after intravitreal MTX treatment are shown in Table 2. The mean IL-6 levels changed from 165.7±87.2, 58.6 pg/mL (mean ± SD, median) to 849.2±785.9, 10.6, pg/mL, but this change was not statistically significant (p = 0.733). IL-10 levels significantly decreased from 5735.8±2123.1, 2132.5 pg/mL to 1755.0±1636.4, 2.9 pg/mL after treatment (p = 0.001). The IL-10-to-IL-6 ratio also decreased from 297.6±112.0, 102.1 to 19.2±12.0, 0.5 (p = 0.003).

### Choroidal vascular structure changes according to treatment

After intravitreal MTX treatment, the mean SFCT, TCA, LA, and SA measurements were significantly decreased, from 290.8±93.5 to 244.4±86.2 μm (p<0.001), 1.30±1.79 to 0.69±0.23 (p = 0.001), 0.98±1.80 to 0.45±0.22 (p = 0.004), and 0.32±0.08 to 0.26±0.06 (p = 0.025), respectively. The mean CVI did not show any significant changes during the remission phase (p = 0.39).

**Fig 2:**
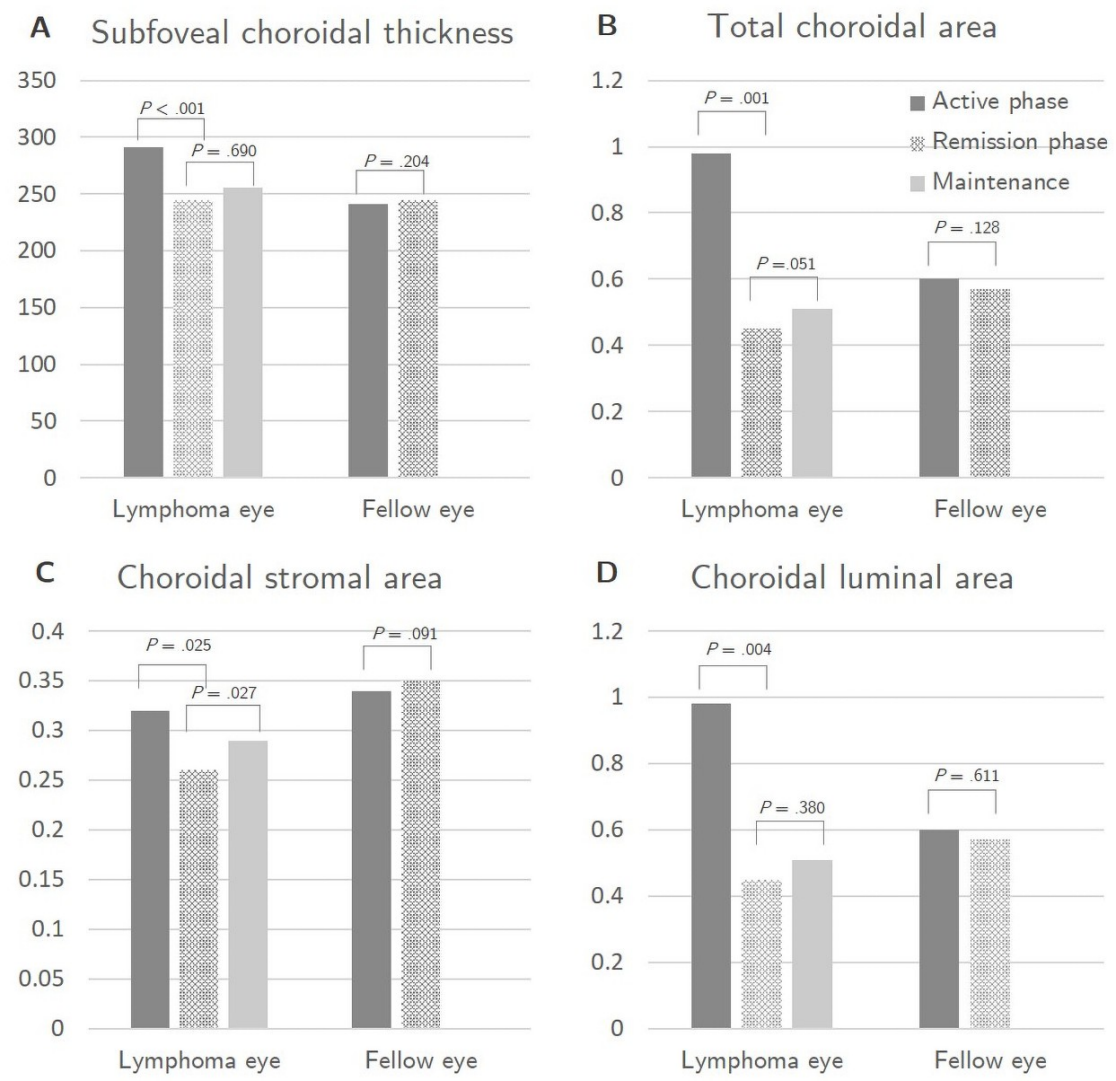
Comparison of choroidal measurements in vitreoretinal lymphoma (VRL) eyes and their fellow eyes. Subfoveal choroidal thickness (SFCT) is shown in μm, and the total choroidal area (TCA), choroidal stromal area (SA), and choroidal luminal area (LA) are shown in mm^2^. Data are expressed as the mean ± standard deviation. (A) In VRL eyes, the mean SFCT was 290.8±93.5 in the active phase and 244.4±86.2 in remission (p<0.001). Mean SFCT after maintenance treatment was 255.50±80.61, which was not significantly different from that in remission (p = 0.690). In the fellow eyes, the mean SFCT was 241.3±100.9 in the active phase and 244.4±103.1 in remission (p = 0.204). (B) In VRL eyes, the mean TCA was 1.30±1.79 in the active phase and 0.69±0.23 in remission (p = 0.001). Mean TCA after maintenance treatment was 0.81±0.31, which was not significantly different from that in remission (p = 0.051). In the fellow eyes, the mean TCA was 0.94±0.37 in the active phase and 0.92±0.36 in remission (p = 0.128). (C) In VRL eyes, the mean SA was 0.32±0.08 in the active phase and 0.26±0.06 in remission (p = 0.025). Mean SA after maintenance treatment was 0.29±0.07, showing a slight increase compared to that in remission (p = 0.027). In the fellow eyes, the mean SA was 0.34±0.12 in the active phase and 0.35±0.13 in remission (p = 0.091). (D) In VRL eyes, the mean LA was 0.98±1.80 in the active phase and 0.45±0.22 in the remission phase (p = 0.004). Mean LA after maintenance treatment was 0.51±0.24, which did not differ significantly from that in remission (p = 0.380). The post-treatment decrease in the choroidal area in VRL patients was more prominent for LA compared to SA, but there was no significant difference in the choroidal vascular index. In the fellow eyes, the mean LA was 0.60±0.25 in the active phase and 0.57±0.23 in remission (p = 0.611).

After maintenance treatment, SFCT, TCA, and LA showed slight increases, but there were no significant differences in the remission phase (p>0.05). Additionally, CVI did not show a significant difference (p>0.05). Only SA showed a significant increase (p = 0.03) (Fig 2).

Excluding the four patients with bilateral VRL, OCT analysis of the fellow eye was performed in seven of the eight remaining patients. One patient with unilateral VRL was excluded from this analysis because suitable quality OCT images were not available from the same time point. None of the seven fellow eyes showed signs of VRL or any other specific ophthalmologic abnormalities. SFCT, TCA, LA, and SA did not show significant changes in the non-treated fellow eyes when measured simultaneously as the treated eye (p>0.05; Table 2).

### Correlation between clinical factors, cytokines, and choroidal structure

In the correlation analysis, pre-and post-treatment SFCT, TCA, LA, and SA values were not significantly correlated with IL-6 and IL-10 levels or the IL-10-to-IL-6 ratio (p>0.05). However, the number of injections required for clinical remission was positively correlated with pre-treatment levels of IL-10 and IL-6 (p<0.001, p = 0.031). In addition, there was a positive correlation between the pre-treatment logMAR BCVA and the number of injections, indicating that more injections were required to achieve clinical regression in patients with worse eyesight before starting injections (p = 0.035; Table 3).

**Table 3:** Correlation between the number of injections required for clinical resolution and other clinical values.

			SFCT	TCA	LA	SA	CVI	BCVA	IL-10	IL-6	IL-10/IL-6 ratio
Number of MTX treatment sessions needed for remission, N	Active	Correlation	-0.291	-0.331	-0.418	0.306	-0.291	0.528	0.628	0.540	-0.186
	phase	*p*-value	0.275	0.210	0.107	0.250	0.274	0.035*	0.009*	0.031*	0.491
	Remission	Correlation	-0.277	-0.288	-0.316	-0.089	-0.394	0.327	-0.219	-0.048	-0.078
	phase	*p*-value	0.299	0.280	0.233	0.744	0.131	0.217	0.416	0.860	0.774

SFCT, subfoveal choroidal thickness; TCA, total choroidal area; LA, luminal area; SA, stromal area; CVI, choroidal vascularity index; BCVA, best-corrected visual acuity; logMAR, minimal angle of resolution; IL, interleukin; MTX, methotrexate.

Pearson’s correlation test was used if one of the factors satisfied normality; Spearmans’s rho test was used when neither of the factors satisfied normality.

*p <0.05 were considered to be statistically significant.

## Discussion

In the current study, we investigated choroidal structural changes in VRL eyes after intravitreal MTX treatment. The TCA, LA, SA, and subfoveal choroidal thickness were significantly decreased in the remission phase compared to the active phase, and the aqueous IL-10 and IL-6 levels and IL-10-to-IL-6 ratios were significantly decreased.

In this study, we used choroidal thickness and OCT binarization measurements to analyze the choroidal structure and differentiate between the vascular luminal and interstitial areas. In addition to clinical improvement, we analyzed aqueous cytokine levels and observed decreases in IL-10 and IL-6 levels in immunological analyses. The changes in choroidal structure observed in the current study were similar to those observed in previous studies but showed slight differences regarding certain details. Cicinelli et al. reported a significant reduction in choroidal thickness after intravitreal rituximab treatment in 18 eyes from nine patients [19]. Similarly, Mariko et al. reported decreased SFCT after intravitreal MTX treatment. In particular, in five eyes with primary intraocular lymphoma, the choroidal and interstitial areas of the choroid were reported to decrease post-treatment [25]. In this study, both the vascular and stromal components of the choroidal structure decreased significantly post-treatment.

Intraocular inflammation from VRL causes retino-choroidal change [26–28]. Notably, an increase in the choroidal luminal area has been reported in the active phase of several forms of uveitis. In the active phase of HLA B27-associated anterior uveitis, there is an increase in choroidal thickness, especially in the luminal area of the choroid structure, which decreases post-treatment. These findings can be considered to result from secondary vascular engorgement of the choroid and disruption of the blood-retinal barrier caused by inflammatory cytokines that are secreted during inflammatory conditions [26, 29]. Similarly, there is a significant post-treatment decrease in the CVI, which represents the LA as a proportion of the TCA, similar to that observed in chorioretinal inflammatory diseases such as multiple evanescent white dot syndrome and Vogt-Koyanagi-Harada syndrome [30, 31].

The increase in SA during the active phase of VRL can be attributed to the recruitment of reactive inflammatory cells towards the choroidal stroma or to direct lymphomatous cell invasion of the choroid [19, 25]. The hyper-reflective foci observed within the choroid on OCT during the active phase are indicative of pathologic changes due to lymphoma affecting not only the vitreous humor and sensory retina but also the choroid [32]. However, in a VRL experiment using a murine model, malignant cells that had been injected intravitreally showed a tendency to invade only as far as the subretinal space after retinal filtration, and only two out of 32 mice in the study showed evidence of choroidal invasion [33]. Thus, further studies are needed regarding choroidal invasion by malignant cells in patients with VRL.

Regarding the remarkable decrease of the LA and SA, in addition to the anti-inflammatory effects of MTX, we cannot exclude the possibility of a localized toxic effect of MTX itself. Intriguingly, when additional maintenance treatment was provided after the remission phase, we did not observe further significant decrease in the choroidal structure relative to the remission phase, indicating that there is a more noticeable decrease in the choroidal area when accompanied by improvement of the lymphoma-related inflammatory reaction. The untreated fellow eyes did not show significant changes in choroidal structure during the observation period, suggesting that systemic MTX or systemic conditions did not have a significant effect on changes in choroidal structure.

The changes in choroidal structural values and cytokine levels followed clinical improvements, but the correlation analysis did not reveal a significant correlation. This could be due to differences in the extent of change in the two values. Cytokine levels showed a larger range of changes among subjects. The change in choroidal structure on the other hand, while significant, was relatively small, which could explain the lack of significance in the linear correlation analyses.

In recent studies, several cytokines such as IL-10 and IL-35 have been reported to be associated with the clinical prognosis of VRL [12, 34]. We found that a high pre-treatment IL-10-to-IL-6 ratio and poor visual acuity were associated with needing a high number of injections to achieve clinical remission. These trends indicate that IL-10 and IL-6 levels are potential indicators of VRL activity or severity. However, IL-10 levels were significantly reduced after treatment, and even if the patient was judged to be in remission based on OCT imaging, fundus examination, or anterior inflammation assessment. Most patients did not show complete negative conversion of IL-10 or the IL-10-to-IL-6 ratio, with some amount of IL-10 still detected. Previously, Harish et al. found a decrease in IL-10 level following intravitreal MTX and rituximab treatment in VRL and suggested that elevated level of IL-10 may point to impending disease recurrence even in clinically quiet VRL [35]. However, there is still insufficient evidence to use negative conversion of specific cytokine levels as a criterion for the remission of VRL. Nevertheless, given that IL-10 is expressed by lymphoma B-cells and IL-6 levels increase in the active phase of ocular inflammatory conditions, even if a VRL patient shows improved clinical findings during treatment, further maintenance therapy should be considered, and the patient’s course should be closely monitored.

This study has several limitations. First, the analysis was based on a relatively small sample size. This is because the prevalence of VRL is not high, and we only analyzed patients who had undergone sampling and for whom a suitable quality of images was available. In addition, to minimize differences due to OCT image capture, we used 1500-μm images in a single scan through the fovea as a representative image. A large area or volume scan over the entire choroid can provide more information. Further, some researchers have reported that choroidal thickness measurements can show diurnal variation or variation along the axial length; however, axial length was not analyzed in our study [36–38]. Previous studies have reported that the CVI is not affected by axial length, and circadian variation is not well established. Nevertheless, it is possible that these factors could have affected our results [24, 39]. Finally, because this study used a retrospective design, it is possible that some bias could have been introduced in the patient enrolment process, and other than the non-treated fellow eye, we were unable to include an intravitreal MTX-treated healthy control group. Furthermore, these limitations need to be addressed in future studies.

In conclusion, eyes with active VRL exhibited choroidal thickening with increased vascular and stromal areas that decreased after remission post intravitreal MTX treatment. Additionally, higher aqueous IL-10 and IL-6 levels and lower visual acuity in the active phase are related to the number of injections required for remission; this should be considered in the treatment of patients with VRL.

## Supporting information

S1 Data (XLSX)
